# FK506 reduces albuminuria through improving podocyte nephrin and podocin expression in diabetic rats

**DOI:** 10.1007/s00011-015-0893-y

**Published:** 2015-11-13

**Authors:** X.-M. Qi, J. Wang, X.-X. Xu, Y.-Y. Li, Y.-G. Wu

**Affiliations:** Department of Nephrology, The First Affiliated Hospital of Anhui Medical University, Hefei, 230022 China

**Keywords:** Diabetic nephropathy, FK506, Podocytes, Nephrin, Podocin, Macrophage

## Abstract

**Objective and design:**

Several works in the setting of early experimental diabetic nephropathy using anti-inflammatory drugs, such as the calcineurin inhibitor FK506, have shown prevention of the development or amelioration of renal injury including proteinuria. The exact mechanisms by which anti-inflammatory drugs lower the albuminuria have not been still clarified well.

**Materials:**

The diabetic rats were induced by using streptozotocin.

**Treatment:**

The diabetic rats were subjected to oral FK506 treatment at a dose of 0.5 or 1.0 mg/kg daily for 4 weeks.

**Methods:**

Renal histology for the ultrastructural evaluation was determined by electron microscope, followed by analyses of renal nephrin and podocin and detection of renal iNOS^+^ macrophages and NF-κB-p-p65^+^.

**Results:**

Elevated 24-h urinary albumin excretion rate was markedly attenuated by FK506 treatment. In diabetic model rats, FK506 treatment at a dose of 0.5 or 1.0 mg/kg significantly increased the expression of nephrin and podocin when compared to control. As expected, rats in control diabetic group had an increase in GBM thickening and foot process effacement when compared to normal rats; increased GBM thickening and foot process effacement were ameliorated by FK506 treatment with 0.5 and 1.0 mg/kg. Histologically, there was marked accumulation of ED-1^+^cells (macrophages) in diabetic kidneys, and FK506 treatment failed to inhibit it. In contrast, FK506 treatment at 0.5 and 1.0 mg/kg doses significantly inhibited the elevated ED-1^+^/iNOS^+^ cells in the kidneys of diabetic rats. ED-1^+^/NF-κB-p-p65^+^ cells were significantly increased in positive diabetic kidneys compared to those of normal rats. FK506 treatment at 0.5 and 1.0 mg/kg significantly attenuated the elevated ED-1^+^/NF-κB-p-p65^+^ cells in diabetic kidneys. Additionally, a positive correlation was observed between ED-1^+^/iNOS^+^ cells and albuminuria (*r* = 0.87, *p* < 0.05). Likewise, ED-1^+^/iNOS^+^ cells were correlated negatively with both nephrin and podocin protein (*r* = −0.70, *p* < 0.05; *r* = −0.68, *p* < 0.05, respectively).

**Conclusion:**

Our results show that FK506 not only upregulates expression of nephrin and podocin but also inhibits macrophage activation to protect against podocyte injury.

## Introduction

Diabetic nephropathy (DN), one of the major serious complications of diabetes mellitus, is the leading cause of end-stage renal disease and high mortality in diabetic patients [1]. The appearance of microalbuminuria is a detectable early marker of DN [2]. Microalbuminuria may develop into severe proteinuria and progressively decline glomerular filtration rate (GFR) [3]. The detailed molecular mechanisms underlying the correlation between albuminuria and DN remain elusive. The pathogenesis of DN involves numerous factors, such as hyperglycemia, advanced glycation end products, poly(ADP-ribose) polymerase activation, protein kinase C, oxidative stress and inflammation [4–6]. Chronic inflammation is closely associated with permeability changes in the glomerular filtration barrier and proteinuria in DN [7]. Podocytes are located at the outer layer of the filtration barrier, and injury to podocytes is involved in the inflammatory processes of DN [8]. Recently, several experimental reports showed the presence of altered nephrin and podocin expression in different models of diabetic nephropathy, such as mice with streptozotocin (STZ)-induced diabetes and non-obese diabetic mice [9, 10]. Previous study demonstrated that activated macrophages produce proinflammatory cytokines and thereby repress expression of nephrin in podocytes, leading to development of proteinuria, which revealed that activated macrophages play a crucial role in the injury of podocytes [11].

Emerging evidence suggests inflammation to play essential roles in the pathogenesis of diabetic complications including DN, and macrophages show a central role in the process. Infiltrating macrophages have been found in both diabetic kidneys from experimental animal models and human DN renal biopsies. However, mounting results tend to indicate that it is the activation state of recruited macrophages, rather than their infiltrating numbers, that finally determines the evolvement and prognosis of renal injury [12]. Direct evidence that macrophages can induce proteinuria comes from adoptive transfer studies in experimental glomerulonephritis [13]. In this model, macrophage-induced proteinuria is dependent upon the state of macrophage activation, with interferon-γ (IFN-γ) activation of macrophages augmenting proteinuria, while glucocorticoids suppress the macrophage pro-inflammatory response and inhibit proteinuria [14].

FK506 is a novel potent immunosuppressant alike cyclosporin A (CsA) and was able to prevent early DN progression through the anti-inflammatory effects [15]. In a previous study, we showed that FK506 beneficially affected the progression of DN, reducing albuminuria in diabetes is associated with inhibit early kidney hypertrophy and ECM expansion in STZ-induced diabetic rats [16]. However, the exact mechanisms by which calcineurin inhibitors lower the albuminuria have not been still clarified well. Therefore, the aim of this study was to test the hypothesis that FK506 attenuates albuminuria through protection against podocyte impairment via regulation of activated macrophages in early experimental diabetic kidney.

## Materials and methods

### Drugs and reagents

FK506 was purchased from Fujisawa Pharmaceutical Co., Ltd (Osaka, Japan), STZ was purchased from Sigma Chemical Co (St. Louis, Mo, USA), microalbumin assay kit was purchased from Exocell Inc (Philadelphia, Pa., USA), and blood glucose assay kits were purchased from the Nanjing Jiancheng Bioengineering Institute (Nanjing, China). Anti-CaN, nephrin, podocin, phosphorylated NF-κB p65 (p-p65) and iNOS polyclonal antibody were purchased from Santa Cruz Biotechnology (Santa Cruz, CA, USA). Mouse anti-macrophage monoclonal (ED-1) antibody was from Boster Biotechnology (Wuhan, China). Horseradish peroxidase (HRP)-conjugated goat anti-rabbit IgG as well as fluorescein isothiocyanate (FITC)-conjugated goat anti-rabbit IgG was from Boster Biotechnology (Wuhan, China). Chemiluminescence kit was from Amersham Life Science (Little Chalfont, UK).

### Animals

Male Munich-Wistar rats (weight 180–200 g) were obtained from the Experimental Animal Center of Anhui Medical University. A research protocol in accordance with the principles was approved by the animal ethics committee of Anhui Medical University. Animals were housed in wire-bottomed cage under a 12-h light/dark cycle. Room temperature (about 24 ± 1 °C) and humidity (about 60 %) were controlled automatically. The rats were allowed free access to standard laboratory chow and tap water.

### Experimental design

After several days of adaptation, the rats were intraperitoneally injected with STZ diluted in citrate buffer 0.1 M (pH 4.0) at a dose of 65 mg/kg following overnight fasting. Two days later, the diabetic state was confirmed by measurement of tail blood glucose levels using a reflectance meter (one touch II, Lifescan LTD, China). Blood glucose levels were measured twice a week. Diabetic rats were then divided into three groups (*n* = 10 per group), avoiding any inter-group differences in blood glucose levels. A control group of rats was also included. The normal and control diabetic group was given 0.5 % sodium carboxymethylcellulose (CMC-Na), while the experiments rats were given orally the FK506 (suspended in 0.5 % CMC-Na) at a dose of 0.5 and 1.0 mg/kg daily using a stomach tube for 4 weeks.

### Blood sample and tissue collection

After treated for 4 weeks, body weight of the rats was measured at the end of the experiment. Rats were then anesthetized by intraperitoneal injection of sodium pentobarbital (50 mg/kg) and placed on a temperature-regulated table. The right jugular artery was catheterized and used for blood sampling. Blood glucose levels were determined with a glucose analyzer. The kidney was perfused in vivo via the abdominal aorta with 100 ml of normal saline at 4 °C. The left renal vein was punctured to permit the perfusate to drain, and the kidney was removed immediately and snap-frozen in isopentane (−70 °C) for subsequent histologic studies. The remaining kidney was stored at −70 °C for Western blotting analysis.

### Urinary albumin excretion

Prior to killing, rats were placed in metabolic cages for collection of urine over 24-h period of time and used for measurement of albumin levels. After centrifugation, aliquots of the supernatant from urine sample were frozen at −70 °C for subsequent analysis of albumin levels by using an enzyme-linked immunoabsorbent assay with an anti-rat albumin antibody. The 24-h albumin excretion was calculated by multiplying the urinary albumin excretion by the 24-h urine volume.

### Renal histology

For the ultrastructural evaluation, the kidney tissue was fixed in 3 % glutaraldehyde, postfixed in 1 % osmium tetroxide, imbued with uranyl acetate and embedded in epoxy resin (epon). The specimen was thin sectioned and examined under a transmission electron microscope (JEM-1200EX II; JEOL, Tokyo, Japan).

Electron micrographs of 5–10 glomeruli per kidney were randomly taken at magnification 1000× and 20,000× for each rat. The mean glomerular basement membrane (GBM) thickness was calculated using the measurements from three different sites in the cross section, with the aid of Image J. Tangentially sectioned GBM was excluded from the analysis. Photomicrographs of the GBM were also analyzed for the density of slit pores between the podocyte foot processes using published methods [17]. The number of slit pores was counted and divided by the GBM length (mm) to arrive at the linear density.

### Protein extraction and Western blot

Kidney tissue samples were homogenized and lysed in an SDS-PAGE sample buffer, boiled and centrifuged, and the supernatant was recovered by centrifuge. The protein concentration was quantified by using a dye binding assay of Bradford, with bovine serum albumin as a standard. Protein samples were then separated by SDS-PAGE, electroblotted onto nitrocellulose membranes, incubated with a blocking buffer for 1 h and then incubated with primary antibody overnight at 4 °C. The membranes were then incubated with a HRP-labeled goat anti-rabbit IgG. The bound secondary antibody was detected by enhanced chemiluminescence. Housekeeping protein β-actin was used as a loading control. Positive immunoreactive bands were quantified densitometrically (Leica Q500IW image analysis system) and expressed as ratio of CaN, nephrin and podocin to β-actin in optical density units.

### Immunohistochemistry

Immunofluorescence analysis of nephrin and podocin in the renal tissue was performed on 4-µm cryostat sections. Sections were fixed in acetone/ethanol (4:1) solution for 10 min and washed in phosphate-buffered saline (PBS). The samples were incubated with 10 % normal goat serum in PBS at room temperature for 1 h and after that with either anti-nephrin antibody or antibody to podocin in 1 % normal goat serum in PBS for 2 h at room temperature. The slides were washed with PBS, and the primary antibody was incubated with FITC-conjugated goat anti-rabbit IgG diluted in 1 % normal goat serum in PBS for 1 h at room temperature. Nephrin and podocin immunoreactivities were analyzed by measuring fluorescence intensity by digital image analysis of images obtained by using a low-light video camera (Beijing Aeronautic and Aerospace University, Beijing, China).

Immunoperoxidase staining for ED-1^+^ macrophages was conducted on 2-µm sections of formalin-fixed renal tissue using antigen retrieval (microwave oven heating in 0.1 M sodium citrate pH 6.0 for 10 min) followed by a three-layer streptavidin–biotin peroxidase complex staining method. Quantitative analysis of ED-1^+^ macrophages in the glomeruli was performed under 400× magnification and expressed as cells/glomerular cross section (gcs). For each section, 20 sequential glomerular profiles were examined. All scoring was performed on blinded slides.

Double immunohistochemical staining to simultaneous detection of iNOS^+^ macrophages and NF-κB-p-p65^+^ macrophages was also performed on formalin-fixed paraffin sections (2 μm thick). Immunoperoxidase staining of ED-1 was performed as described above. After development with DAB, the sections were placed in 500 ml of 0.1 M sodium citrate buffer (pH 6.0) and microwave-treated for 10 min and then incubated sequentially with 10 % normal goat serum for 10 min followed by anti-iNOS antibody (1:100) and anti-NF-κB-p-p65 antibody (1:100) overnight at 4 °C. The sections were subsequently incubated with alkaline phosphatase-labeled goat anti-rabbit IgG antibody and developed with AP-Red to produce a red color. The method for calculating positive double-stained cells is described above.

### Statistical analysis

Data were expressed as the mean ± SEM unless otherwise specified. One-way analysis of variance with pairwise comparisons according to the Tukey method was performed. Since urinary albumin excretion rate followed a non-normal distribution, log transformation analysis was performed prior to statistical analysis of this parameter. Differences were considered significant if the *P* value was less than 0.05. Correlation analysis was calculated using the Spearman rank-order correlation, and the *p* value <0.05 was considered to be statistically significant.

## Results

### The effects of FK506 on the clinical characteristics of the diabetic rats

The positive control diabetic rats showed to have increased blood glucose levels. However, there were no effects on blood glucose observed in FK506-treated rats. The ratio of kidney weight to body weight (relative kidney weight) was significantly higher in control diabetic than that in normal rats. FK506 treatment with 1.0 mg/kg for 4 weeks significantly reduced relative kidney weight in diabetic rats. In positive control diabetic rats, albuminuria was significantly increased when compared to normal rats, whereas FK506 treatment at 0.5 and 1.0 mg/kg markedly attenuated the increase in albuminuria from the diabetic rats, although the level was still higher than that of rats (Table 1).

**Table 1:** Clinical and biochemical parameters in four groups of rats

Group	Glu (mmol L^−1^)	BW (g)	KW/BW (g/100 g)	UAER^a^ (mg·24 h^−1^)
Normal	6.88 ± 0.81	351.6 ± 15.40	0.30 ± 0.04	0.29×/÷1.1
Control diabetic	26.80 ± 2.98**	224.2 ± 13.20**	0.51 ± 0.04*	0.95×/÷1.2**
Control diabetic + FK506 0.5 mg/kg	27.10 ± 2.44**	227.8 ± 12.64**	0.46 ± 0.05*	0.78×/÷1.1*^,#^
Control diabetic + FK506 1.0 mg/kg	27.22 ± 2.35**	258.2 ± 18.87**	0. 34 ± 0.06^#^	0.55×/÷1.1**^,##^

Data are expressed as mean ± SEM. Number of rats in each group was 10. A shown as geometric mean tolerance factor

*Glu* glucose, *BW* body weight, *KW/BW* kidney-to-body weight ratio, *UAER* urinary albumin excretion rate

* *p* < 0.05 compared with normal group; ** *p* < 0.01 compared with normal group; ^#^ *p* < 0.05 compared with control diabetic group; ^##^ *p* < 0.01 compared with control diabetic group

### FK506 recovers GBM thickening and podocyte foot process in diabetes kidney

**Fig. 1:**
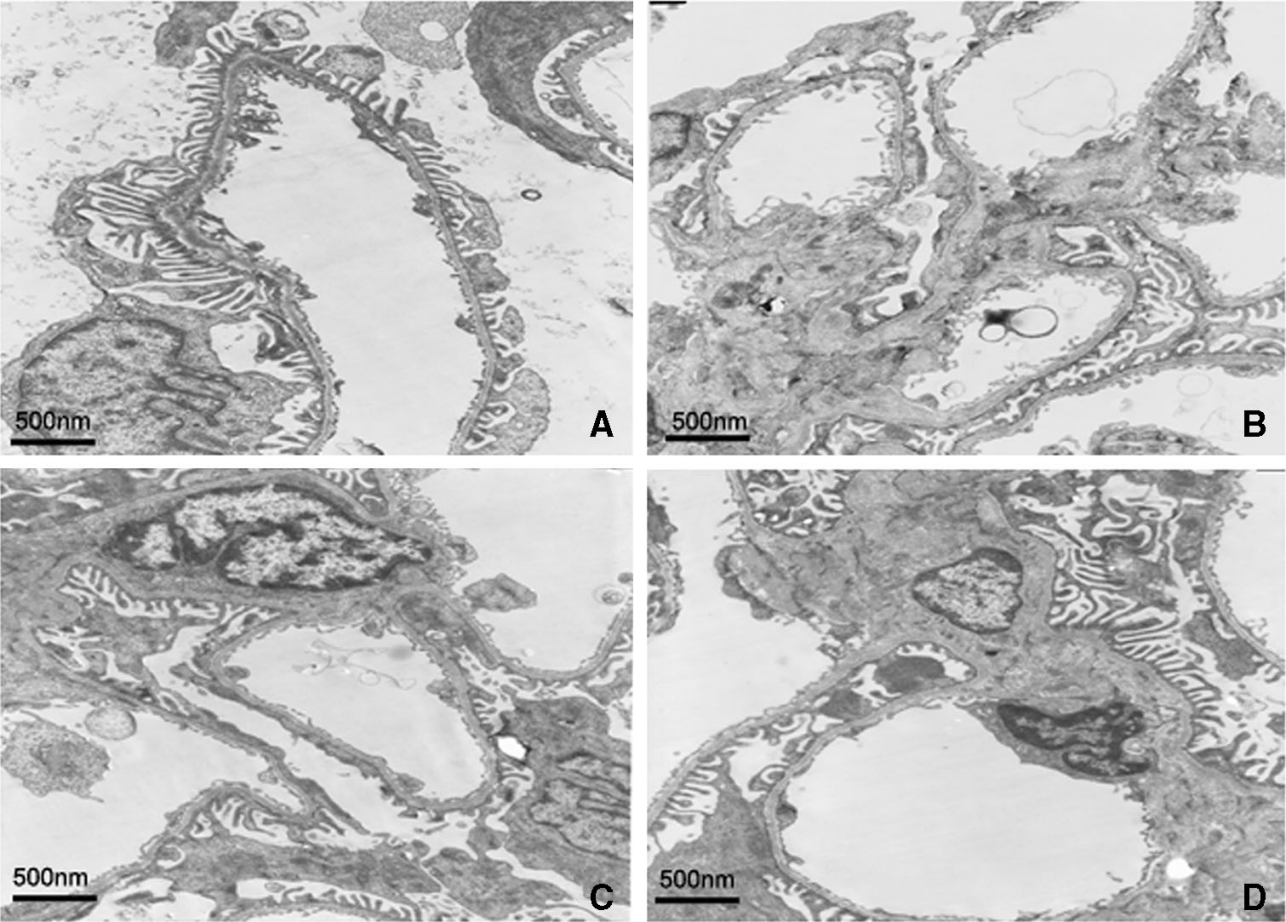
Representative micrographs of kidney tissue-stained electron micrograph of a glomerulus from normal rats (**a**), control diabetic (**b**), control diabetic + FK506 0.5 mg/kg (**c**) and diabetic + FK506 1.0 mg/kg (**d**). Magnification ×5000

The kidney ultrastructure was further examined by electron microscopy. As shown in Fig. 1 and Table 2, normal morphology of the glomerular filtration barrier including GBM and podocyte foot process was seen in normal rats. As expected, rats in control diabetic group had an increase in GBM thickening and foot process effacement when compared to normal rats, increased GBM thickening and foot process effacement were ameliorated by FK506 treatment with 0.5 and 1.0 mg/kg.

**Table 2:** Glomerular ultrastructure parameters in four groups of rats

Group	Foot process width (μm)	Foot process fusion rate (%)	GBM thickness (μm)
Normal	0.27 ± 0.03	2.12 ± 0.36	0.26 ± 0.03
Control diabetic	0.77 ± 0.03*	75.33 ± 14.61*	0.71 ± 0.06*
Control diabetic + FK506 0.5 mg/kg	0.43 ± 0.02*^,#^	35.24 ± 6.07*^,#^	0.42 ± 0.03*^,#^
Control diabetic + FK506 1.0 mg/kg	0.41 ± 0.02*^,#^	33.39 ± 7.48*^,#^	0.40 ± 0.01*^,#^

* *p* < 0.05 compared with normal group; ^#^ *p* < 0.05 compared with control diabetic group

### FK506 inhibits CaN expression in diabetic kidney

**Fig. 2:**
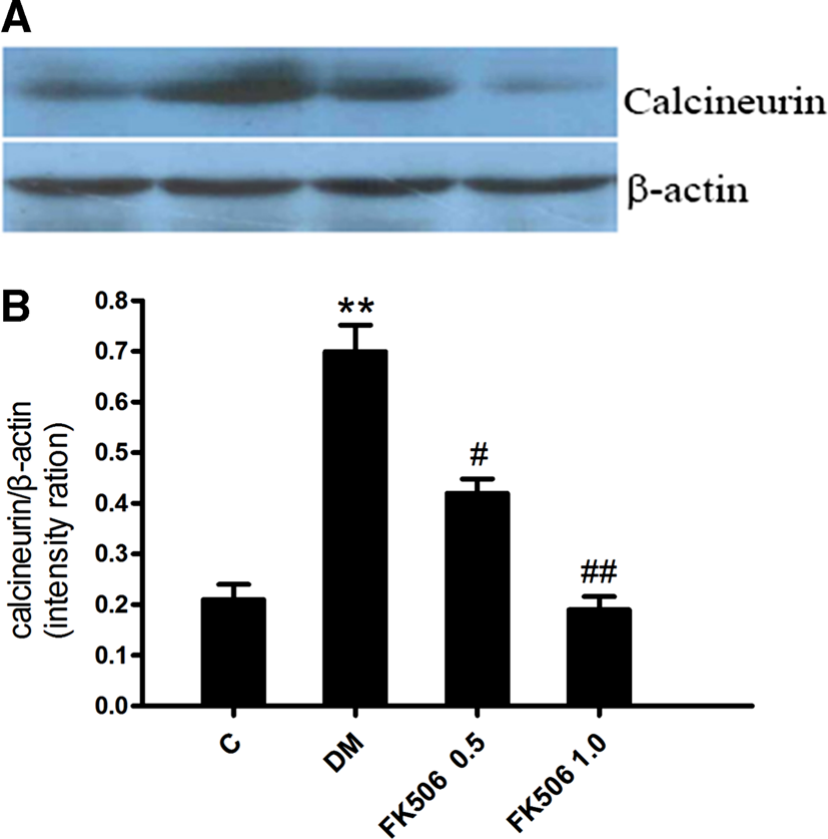
Western blot analysis of calcineurin protein (*upper panel*) and densitometric analysis (*lower panel*) in renal tissue from normal, control diabetic, diabetic + FK506 0.5 mg/kg and diabetic + FK506 1.0 mg/kg. Values are the mean ± SEM. *n* = 10, **p < 0.01 versus C, ^#^ *p* < 0.05, ^*##*^ *p* < 0.01 versus DM

To detect molecular changes in these experimental rats, we performed Western blot analysis of renal CaN expression (Fig. 2). The data showed an increase in amount of immunoreactive peptide in the kidney of positive control diabetic rats compared to that of normal rats. Specifically, densitometric analysis of the Western blot showed a 2.4-fold increase in the amount of CaN in positive control diabetic rats compared to the normal rats. In contrast, FK506 treatment at 0.5 and 1.0 mg/kg reduced levels of CaN protein by 38.0 and 73.2 %, respectively.

### FK506 recovers nephrin expression in diabetic kidney

**Fig. 3:**
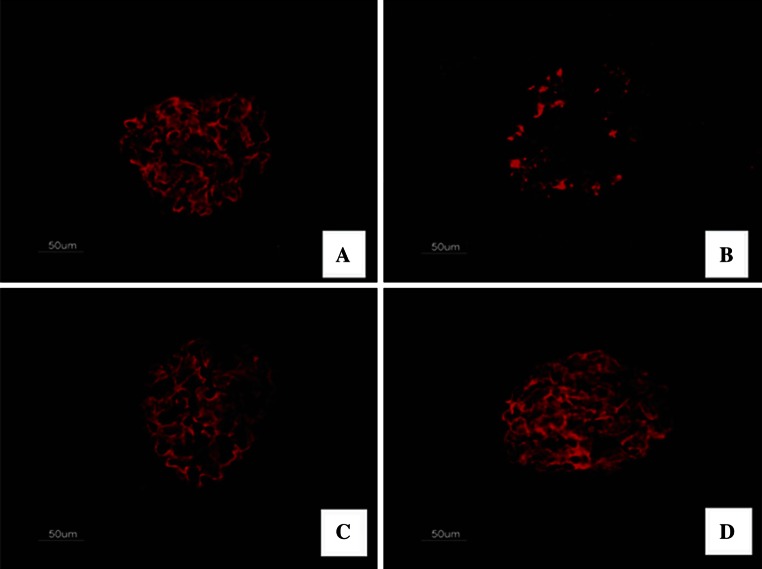
Immunofluorescence staining for nephrin. The representative fields show glomerular expression of nephrin as indicated from normal rats (**a**), control diabetic (**b**), control diabetic + FK506 0.5 mg/kg (**c**) and diabetic + FK506 1.0 mg/kg (**d**). Magnification ×400

To further reveal the mechanism responsible for the prevention in albuminuria in FK506-treated diabetic rats, we studied the expression of nephrin, a key protein of the glomerular slit membrane. We first assessed the expression of nephrin by immunohistochemistry. As shown in Fig. 3, there was a finely dotted linear epithelial staining in the normal group glomeruli. In contrast, the staining of glomeruli from untreated diabetic rats was attenuated, more dispersed and clustered. Importantly, this diabetic-induced loss of glomerular nephrin expression was to a large degree prevented by FK506 treatment with 0.5 and 1.0 mg/kg.

**Fig. 4:**
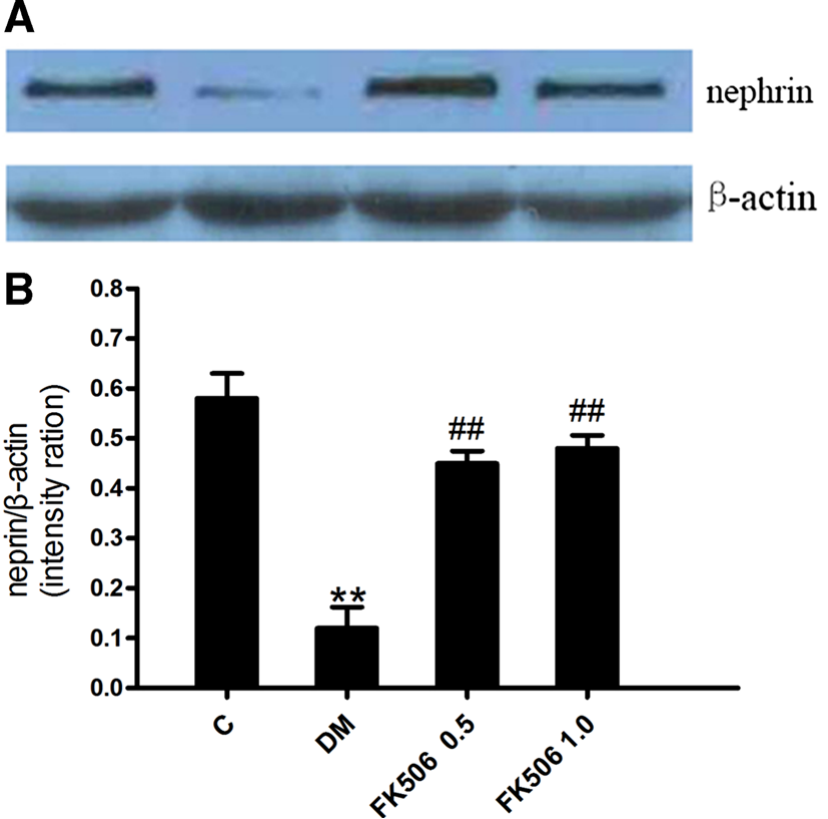
Western blot analysis of nephrin protein (*upper panel*) and densitometric analysis (*lower panel*) in renal tissue from normal, control diabetic, diabetic + FK506 0.5 mg/kg and diabetic + FK506 1.0 mg/kg. Values are the means ± SEM. *n* = 10, **p < 0.01 vs C, ^#^ *p* < 0.05, ^*##*^ *p* < 0.01 vs DM

As we observed a prevention of the diabetic-induced nephrin loss by FK506, we next measured the nephrin expression by Western blotting analysis to confirm and quantitatively analyze the results obtained from the immunohistological evaluation. The results are shown in Fig. 4; nephrin was expressed as a single band. Hyperglycemia of 4 weeks reduced the expression of nephrin significantly as observed with immunohistochemical analysis. Diabetic-induced nephrin loss is again prevented in FK506-treated rats.

### FK506 recovers podocin expression in diabetic kidney

**Fig. 5:**
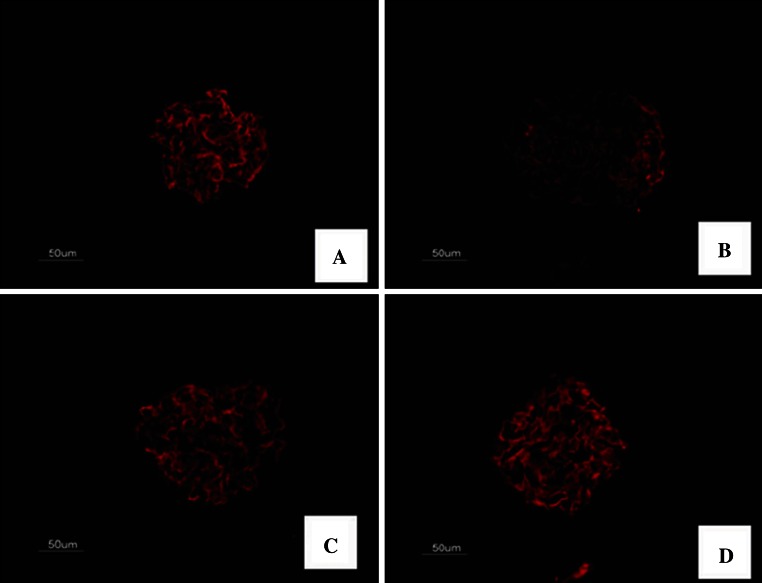
Immunofluorescence staining for podocin. The representative fields show glomerular expression of podocin as indicated from normal rats (**a**), control diabetic (**b**), control diabetic + FK506 0.5 mg/kg (**c**) and diabetic + FK506 1.0 mg/kg (**d**). Magnification ×400

To explore the role of other slit diaphragm proteins, we secondly performed tissue analysis of other slit diaphragm proteins, podocin. The staining of podocin was revealed to form a linear pattern along the glomerular capillary wall in the normal group glomeruli. Notably, the glomerular expression of podocin was significantly reduced in the diabetic rats and was barely detectable, treatment with FK506 moderately but significantly reduced the disappearance of podocin from the glomeruli of diabetic rats (Fig. 5).

**Fig. 6:**
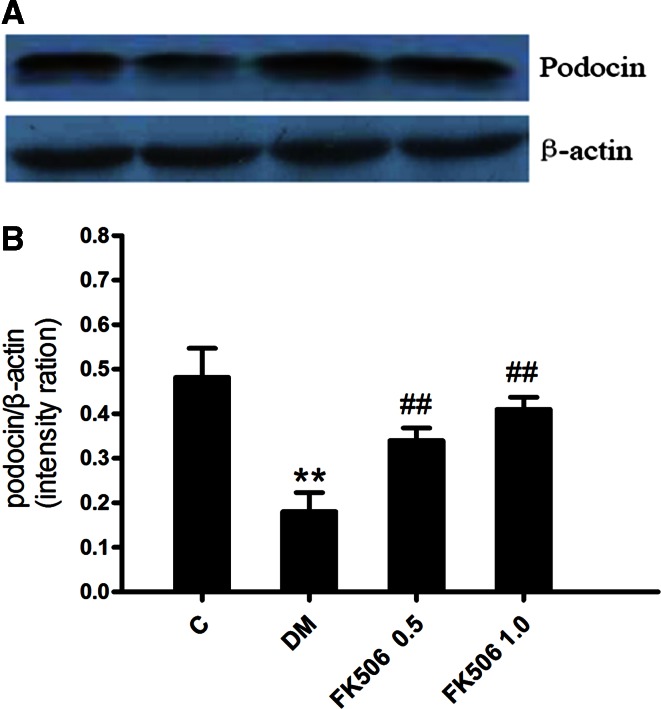
Western blot analysis of podocin protein (*upper panel*) and densitometric analysis (*lower panel*) in renal tissue from normal, control diabetic, diabetic + FK506 0.5 mg/kg and diabetic + FK506 1.0 mg/kg. Values are the means ± SEM. *n* = 10, **p < 0.01 vs C, ^#^ *p* < 0.05, ^*##*^ *p* < 0.01 vs DM

As we observed a prevention of the diabetic-induced podocin loss by FK506, we next measured the podocin expression by Western blotting analysis to confirm and quantitatively analyze the results obtained from the immunohistological tissue evaluation. Western blotting analysis of podocin is shown in Fig. 6. Diabetic-induced podocin loss is also prevented by FK506 treatment with 0.5 and 1.0 mg/kg.

### FK506 inhibits macrophage activation in diabetic kidney

**Fig. 7:**
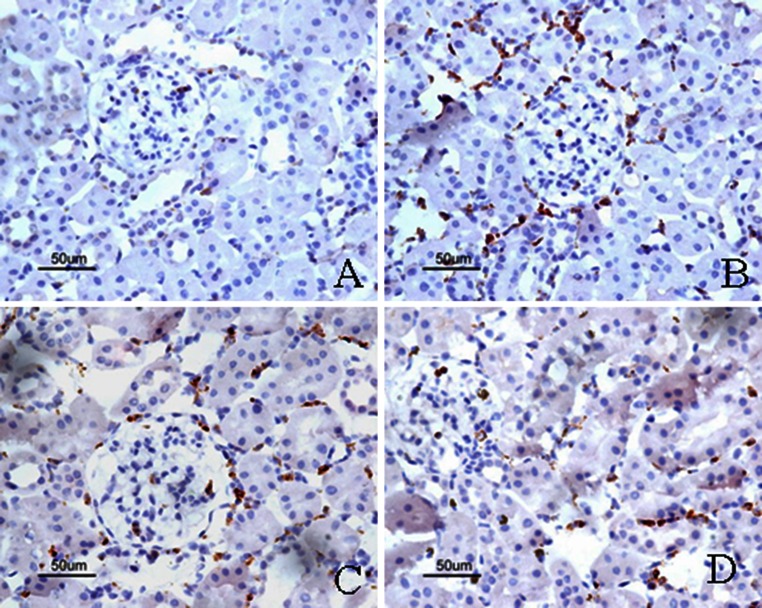
Immunostaining of ED-1. The representative fields show renal expression of ED-1 as indicated, in normal rats (**a**), control diabetic (**b**), control diabetic + FK506 0.5 mg/kg (**c**) and diabetic + FK506 1.0 mg/kg (**d**). Magnification ×400

**Fig. 8:**
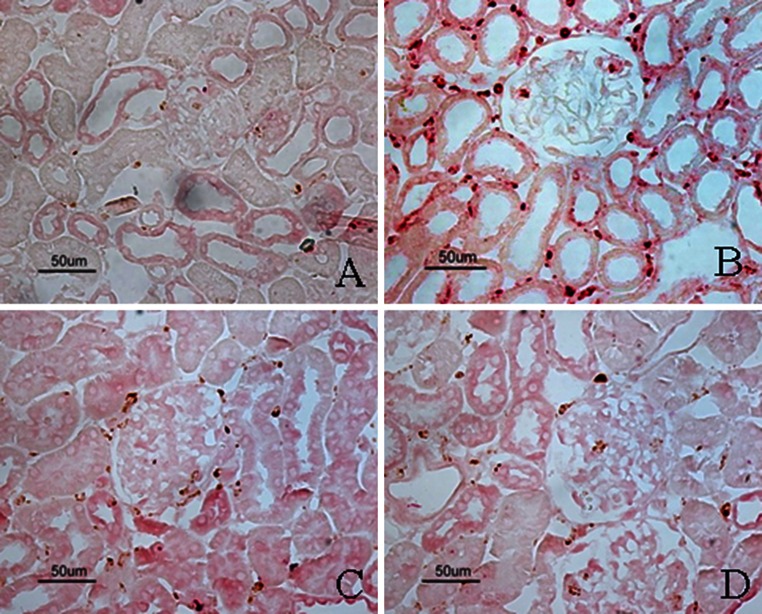
Double immunostaining of ED-1/iNOS. The representative fields show renal expression of ED-1/iNOS as indicated, in normal rats (**a**), control diabetic (**b**), control diabetic + FK506 0.5 mg/kg (**c**) and diabetic + FK506 1.0 mg/kg (D). Magnification ×400

To assess the potential effect of FK506 on renal inflammation, we examined renal infiltration of the ED-1 positive macrophages in STZ-induced DN rats. As shown in Fig. 7, compared with control rats, DN rats exhibited significant macrophages infiltration in the glomeruli at week 4 after STZ induction. However, FK506-treated rats did not show reduction in macrophage infiltrations. In addition, ED-1^+^/iNOS^+^ cells were also significantly increased in the kidneys from positive control diabetic rats but were significantly suppressed by FK506 treatment (Fig. 8; Table 3).

**Table 3:** Semiquantitative assessment of ED-1, ED-1^+^/NF-κB-p-p65^+^ and ED-1^+^/iNOS^+^ immunohistochemistry staining in the glomeruli from four groups of rats

Group	ED-1 (cells/gcs)	ED-1^+^/NF-κB-p-p65^+^ (cells/gcs)	ED-1^+^/iNOS^+^ (cells/gcs)
Normal	0.93 ± 0.70	0.91 ± 0.52	0.67 ± 0.46
Control diabetic	3.27 ± 1.22**	3.92 ± 1.76*	2.07 ± 0.59*
Control diabetic + FK506 0.5 mg/kg	2.80 ± 1.26**	1.60 ± 0.93*^,##^	1.53 ± 0.48**
Control diabetic + FK506 1.0 mg/kg	2.73 ± 1.33**	1.20 ± 0.63*^,##^	1.20 ± 0.56**

gcs was indicated glomerular cross-sectional area. Data are expressed as mean ± SEM. Number of rats in each group was 10

* *p* < 0.05 compared with normal group; ** *p* < 0.01 compared with normal group; ^##^ *p* < 0.01 compared with control diabetic group

### FK506 inhibits NF-κB-p-p65^+^ macrophages in diabetic kidney

**Fig. 9:**
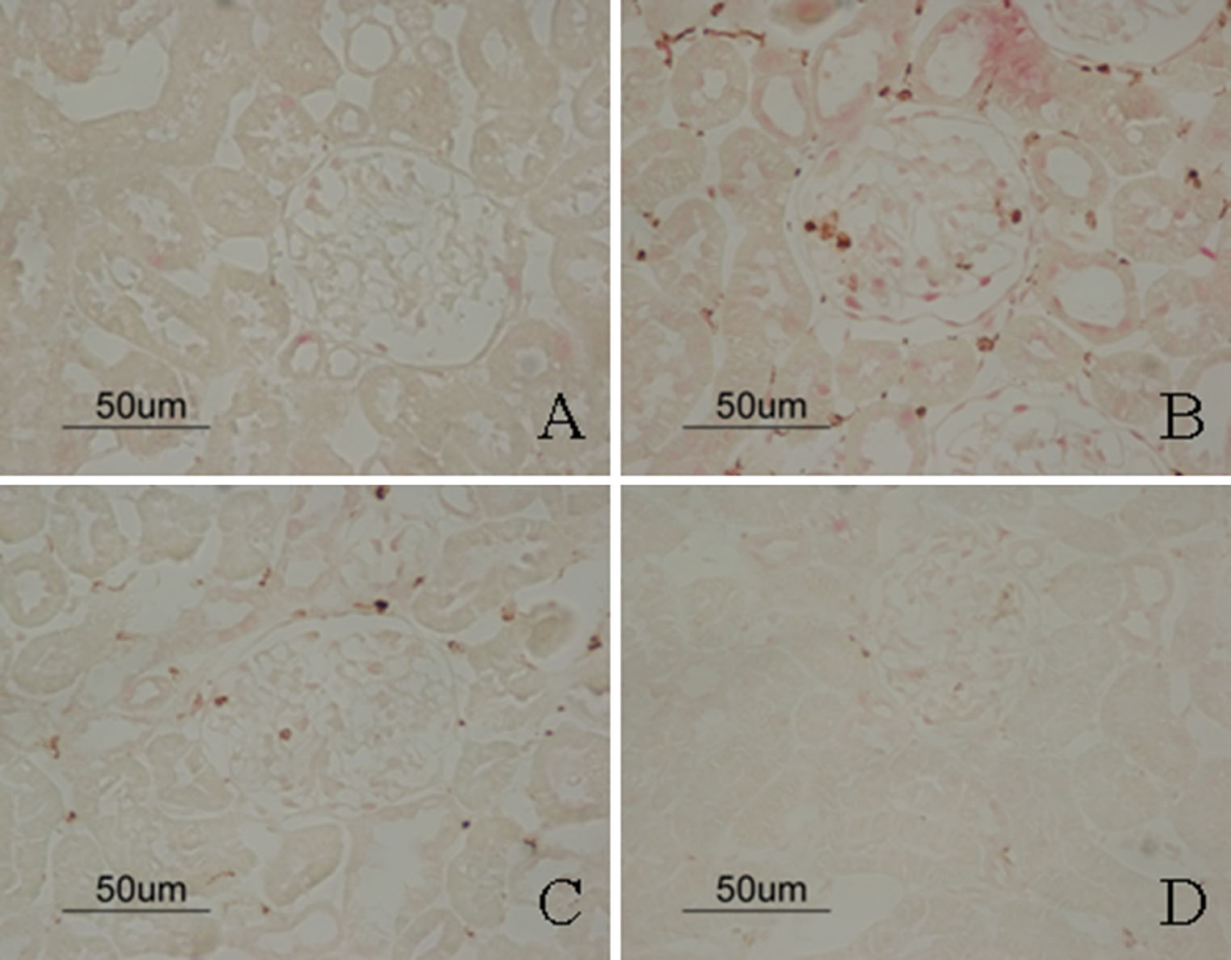
Double immunostaining of ED-1/NF-κB-p-p65. The representative fields show renal expression of ED-1/NF-κB-p-p65 as indicated, in normal rats (**a**), control diabetic (**b**), control diabetic + FK506 0.5 mg/kg (**c**) and diabetic + FK506 1.0 mg/kg (**d**). Magnification ×400

ED-1^+^/NF-κB-p-p65^+^ cells were significantly increased in positive diabetic kidneys compared to those of normal rats. FK506 treatment at 0.5 and 1.0 mg/kg significantly attenuated the elevated ED-1^+^/NF-κB-p-p65^+^ cells in diabetic kidneys (Fig. 9; Table 3).

### Correlation between activated macrophages and albuminuria, nephrin and podocin protein

**Fig. 10:**
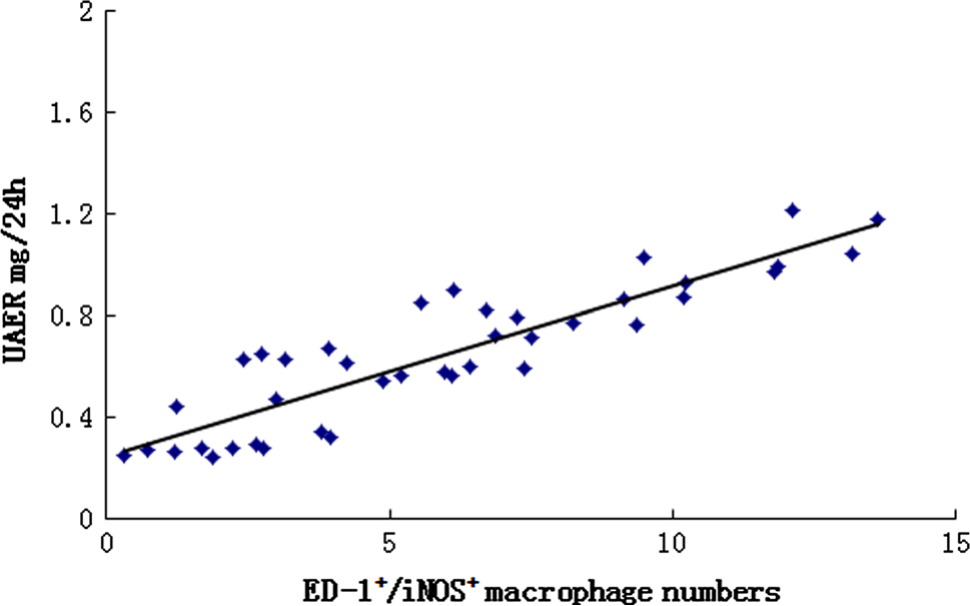
Plot of correlation between UAER (urinary albumin excretion rate) and ED-1^+^/iNOS^+^ macrophage numbers (*r* = 0.87, *p* < 0.05)

As shown in Fig. 10, we found the highest correlation between ED-1^+^/iNOS^+^ cells with albuminuria (*r* = 0.87, *p* < 0.05). Likewise, ED-1^+^/iNOS^+^ cells showed negative correlations with nephrin and podocin protein (*r* = −0.70, *p* < 0.05; *r* = −0.68, *p* < 0.05, respectively).

## Discussion

DN is one of the most important health problems worldwide, and this is likely to worsen to critical levels in the next decades [1]. Recently, increasing evidence has been obtained demonstrating that podocytes play an important role in the pathogenesis of proteinuria and progression of DN [18]. The present study shows that 4 weeks after induction of diabetes, marked albuminuria associated with reduction in podocyte-specific protein nephrin and podocin is established. Furthermore, macrophages recruitment and activation in the glomeruli were increased in diabetic rats. FK506 treatment showed significantly increased expression of nephrin and podocin when compared to control. FK506 treatment results in the inhibited macrophages activation.

The glomerular podocyte is known to play a critical role in kidney structure and urinary filtration. The podocyte foot process surrounds the outside of the GBM and the gaps between adjacent podocyte foot processes form slit diaphragms (filtration slits). A series of recent studies have suggested that podocyte injury plays a key role in the development of DN [19, 20]. Drugs that have beneficial effects on podocytes can improve our ability to treat DN [21]. In the present study, its was shown that after treatment with FK506, the foot process effacement and GBM thickening ameliorated, suggesting the protective effects of FK506 against podocyte injury.

Finally, we examined the mechanism underlying the anti-albuminuric effect of FK506 agonist in diabetes. Podocyte slit diaphragm plays a critical role in the maintenance of normal barrier function of glomerular capillary wall. The dysfunction of podocyte slit diaphragm causes proteinuria [22]. As two key functional molecules in podocyte slit diaphragm, nephrin is located at the outer leaflet of plasma membranes of podocyte slit diaphragm, while podocin is the interaction with nephrin [23]. Recently, we and other studies demonstrated that the alteration in nephrin and podocin is involved in the development of proteinuria in several models of DN [9]. The anti-proteinuric effect of 1, 25-dihydroxyvitamin D3 has been shown to be closely associated with the preservation of nephrin and podocin expression in STZ-induced DN rats [24]. Therefore, the abnormal expression and distribution of nephrin and podocin could be a mechanism and a therapeutic target for proteinuria in DN.

FK506 is mainly used as an immunosuppressant in allogeneic organ transplants and autoimmune diseases. Binding of FK506 to its specific intracellular receptor (FK506 binding protein) inhibits calmodulin activity. Clinical observations have found that it can also effectively reduce renal damage and proteinuria in glomerular diseases such as MCN, FSGS, MsPGN and MPGN [25, 26]. Our previous studies documented that FK506 ameliorated albuminuria and inhibit early kidney hypertrophy and ECM expansion in STZ-induced diabetic rats, which was possibly achieved through the suppression of the expression of several injurious cytokines in the glomeruli [16]. However, we still do not fully understand how albuminuria was reduced by FK506 in this model. To investigate whether the anti-albuminuria effect of FK506 could be related to the improvement in podocyte slit diaphragm function, we compared the distribution and expression of nephrin and podocin at protein levels in glomeruli on 4 weeks after STZ-induced diabetic rats between FK506 and vehicle groups. In our study, diabetic kidney nephrin and podocin expression was significantly downregulated compared with normal rats. Interestingly, the treatment of FK506 not only decreased albuminuria, but also recovered the distribution and expression of nephrin and podocin in glomeruli.

Macrophage infiltration is a common feature in both human and experimental renal diseases [27]. These infiltrating cells are strongly associated with the severity of kidney injury and progressive chronic renal failure. In experimental crescentic glomerulonephritis, inhibition of macrophages infiltration in glomerular significantly alleviated crescent formation and proteinuria [28]. In our study, STZ-induced DN rats were clearly characterized by large macrophage infiltration in the glomeruli and interstitium at the early stage after disease induction, which was followed by the development of podocyte injury and profound albuminuria, suggesting macrophages as a major constituent of the infiltrated inflammatory cells in DN.

Although commonly recognized for the pathogenic role of macrophage in renal inflammation and fibrosis, macrophages also play a critical role in tissue remodeling and repair, as well as in immune regulation. However, macrophage infiltration into the kidney is not always pathogenic because only activated macrophages can produce injury [29]. Recently, using conditionally immortalized reporter podocytes, Takno et al. [30] found that bystander macrophages as well as macrophage-derived cytokines IL-1β and TNF-α markedly suppressed activity of the nephrin gene promoter in podocytes. The cytokine-initiated repression was reversible, observed on both basal and inducible expression, independent of Wilms’ tumor suppressor WT1 and caused in part via activation of the phosphatidylinositol-3-kinase/Akt pathway. Activated macrophages produce inflammatory cytokines abundantly, which is involved in the development of proteinuria in glomerular disease [31]. The requirement for macrophage activation to induce podocyte damage is consistent with experimental studies in which the ability of adoptively transferred macrophages to induce renal injury is dependent upon their activated status. Hanning et al. [32] cocultured podocytes and bone marrow-derived monocyte/macrophage subsets in a transwell system. Data show that iNOS^+^ macrophages, but not iNOS^−^ macrophages, induced podocyte injury. In additional, studies using RAW 264.7 macrophage cells have shown that expression of inflammatory factors was induced by calcineurin-activated processes resulting from activation of NF-κB and suppressed by the calcineurin inhibitor FK506 [33]. In this study, we observed a significant increase in the number of ED-1^+^/iNOS^+^ cells and ED-1^+^/NF-κB-p-p65^+^ cells in the STZ rat model; this was, however, abrogated by FK506 administration. We were also able to further confirm the strong positive correlation between ED-1^+^/iNOS^+^ cells and albuminuria and ED-1^+^/iNOS^+^ cells negative correlations with nephrin and podocin protein. These results indicate that in the STZ model of rat podocyte injury, nephrin and podocin levels are reduced, likely contributing to increased ED-1^+^/iNOS^+^ cells. Extrapolation of these findings to the in vivo setting requires caution, but we propose that the major mechanism of calcineurin action is related to the upregulation of the NF-κB-p-p65 in the activated macrophage.

In conclusion, podocyte slit diaphragm dysfunction, such as the disordered distribution and downregulation of nephrin and podocin expression, is critically involved in the pathogenesis of in STZ-induced DN rats. The restoration of the distribution and expression of nephrin and podocin by FK506 could be an important mechanism by which FK506 ameliorates podocyte slit diaphragm dysfunction and macrophages activation. However, the current study is just a proof-of-principle and more studies are needed to mechanistically investigate macrophage infiltration, activation and functions in DN.
